# Observational study to characterise 24-hour COPD symptoms and their relationship with patient-reported outcomes: results from the ASSESS study

**DOI:** 10.1186/s12931-014-0122-1

**Published:** 2014-10-21

**Authors:** Marc Miravitlles, Heinrich Worth, Juan José Soler Cataluña, David Price, Fernando De Benedetto, Nicolas Roche, Nina Skavlan Godtfredsen, Thys van der Molen, Claes-Göran Löfdahl, Laura Padullés, Anna Ribera

**Affiliations:** Pneumology Department, Hospital Universitari Vall d’Hebron, Ciber de Enfermedades Respiratorias (CIBERES), P. de la Vall d’Hebron, 119–129, Barcelona, Spain; Medical Department I, Fürth Hospital, Fürth, Germany; Servicio de Neumología, Hospital Arnau de Vilanova, Valencia, Spain; Centre of Academic Primary Care, University of Aberdeen, Aberdeen, UK; Pneumology Unit, Ospedale Clinicizzato SS. Annunziata, Chieti, Italy; Cochin Hospital, Paris Descartes University, Paris, France; Department of Respiratory Medicine, Bispebjerg University Hospital, Copenhagen, Denmark; University of Groningen, University Medical Center Groningen, Groningen, The Netherlands; Department of Respiratory Medicine and Allergology, Lund University Hospital, Lund, Sweden; Medical Affairs, Almirall, Barcelona, Spain

**Keywords:** Anxiety, ASSESS, COPD, Depression, Dyspnoea, Health status, Observational, Relationship, Sleep quality, Symptoms

## Abstract

**Background:**

Few studies have investigated the 24-hour symptom profile in patients with COPD or how symptoms during the 24-hour day are inter-related. This observational study assessed the prevalence, severity and relationship between night-time, early morning and daytime COPD symptoms and explored the relationship between 24-hour symptoms and other patient-reported outcomes.

**Methods:**

The study enrolled patients with stable COPD in clinical practice. Baseline night-time, early morning and daytime symptoms (symptom questionnaire), severity of airflow obstruction (FEV_1_), dyspnoea (modified Medical Research Council Dyspnoea Scale), health status (COPD Assessment Test), anxiety and depression levels (Hospital Anxiety and Depression Scale), sleep quality (COPD and Asthma Sleep Impact Scale) and physical activity level (sedentary, moderately active or active) were recorded.

**Results:**

The full analysis set included 727 patients: 65.8% male, mean ± standard deviation age 67.2 ± 8.8 years, % predicted FEV_1_ 52.8 ± 20.5%.

In each part of the 24-hour day, >60% of patients reported experiencing ≥1 symptom in the week before baseline. Symptoms were more common in the early morning and daytime versus night-time (81.4%, 82.7% and 63.0%, respectively). Symptom severity was comparable for each period assessed. Overall, in the week before baseline, 56.7% of patients had symptoms throughout the whole 24-hour day (3 parts of the day); 79.9% had symptoms in ≥2 parts of the 24-hour day. Symptoms during each part of the day were inter-related, irrespective of disease severity (all p < 0.001).

Early morning and daytime symptoms were associated with the severity of airflow obstruction (p < 0.05 for both). Night-time, early morning and daytime symptoms were all associated with worse dyspnoea, health status and sleep quality, and higher anxiety and depression levels (all p < 0.001 versus patients without symptoms in each corresponding period). In each part of the 24-hour day, there was also an association between symptoms and a patient’s physical activity level (p < 0.05 for each period).

**Conclusions:**

More than half of patients experienced COPD symptoms throughout the whole 24-hour day. There was a significant relationship between night-time, early morning and daytime symptoms. In each period, symptoms were associated with worse patient-reported outcomes, suggesting that improving 24-hour symptoms should be an important consideration in the management of COPD.

**Electronic supplementary material:**

The online version of this article (doi:10.1186/s12931-014-0122-1) contains supplementary material, which is available to authorized users.

## Background

Despite being preventable and treatable, chronic obstructive pulmonary disease (COPD) is associated with considerable morbidity and mortality [1,2] and its prevalence is expected to increase in the coming decades [3]. The characteristic symptoms of COPD include breathlessness, cough and increased sputum production and, based on cohort studies, there is now extensive evidence that COPD symptoms have a considerable impact on patients’ daily activities, health status and quality of life [4-8]. Furthermore, while COPD is diagnosed clinically based on persistent airflow limitation, it is the impact of symptoms on patients’ daily lives that generally drives them to seek a diagnosis. The importance of considering COPD symptoms in the overall assessment of COPD, and in determining appropriate treatment approaches, is now recognised [9]. Reducing symptoms, improving health status and increasing physical activity are major goals in the management of stable COPD [9].

COPD symptoms have been reported to vary throughout the day [10-12]. In cohort studies, patients with COPD who were receiving ongoing treatment with their normal COPD medication reported that their symptoms were worst in the morning [10,12]. Morning symptoms impact on patients’ normal activities [8,10,12,13] and have been demonstrated to be associated with worse health status and a higher risk of COPD exacerbations [8,13]. In the working population, morning symptoms were also significantly associated with increased annual absenteeism [13]. With regard to night-time symptoms, a recent real-world study also demonstrated that patients with night-time symptoms had significantly worse health status, more sleep disturbances and higher healthcare resource utilisation than patients without night-time symptoms [7].

In a pan-European, observational study, patients’ perception of the variability of their breathlessness was associated with both the severity of breathlessness and frequent exacerbations [10], while the pattern of COPD symptom variability has been shown to be influenced by disease severity [12]. Previous studies have also shown an association between morning or night-time symptoms and reduced lung function [7,13,14]. However, the association between symptoms in each part of the 24-hour day and the severity of airflow obstruction and the inter-relationship between 24-hour COPD symptoms has not previously been investigated in a single patient cohort.

In this observational study, we investigated the prevalence and severity of night-time, early morning and daytime symptoms in patients with stable COPD being treated in clinical practice and explored the relationship between symptoms in each part of the 24-hour day. Additionally, to better understand the relationship between 24-hour symptoms and other aspects of a patient’s overall well-being, we assessed their association with the severity of airflow obstruction and other patient-reported outcomes, including self-perceived dyspnoea, health status, anxiety and depression levels, sleep quality and physical activity level.

## Methods

### Study design

This was a multinational, non-interventional, observational study conducted in 85 clinical practice centres (pulmonologists outpatients and primary care) across Denmark, France, Germany, Italy, The Netherlands, Spain, Sweden and UK (see Additional file 1 for a list of investigators). Patients who met the eligibility criteria were identified consecutively at each site, with each site having a maximum quota to minimise selection bias. The study consisted of a baseline visit (Day 1) and a follow-up telephone interview after 6 months. There were no interventions beyond routine clinical care delivered at the discretion of the physician.

A steering committee, comprising the study country co-ordinators, was involved in the design of the study. The protocol was approved by all necessary ethics committees, as required by law for each country, before study initiation (see Additional file 2 for a list of approval authorities for each country). All patients provided written informed consent.

### Study population

Patients were aged ≥40 years with mild to very severe COPD according to the Global Initiative for Chronic Obstructive Lung Disease (GOLD) spirometric classification [15], (spirometry data from the year before baseline were considered valid). Patients were current or former smokers with a smoking history of ≥10 pack-years and had no history of COPD exacerbation in the previous month.

Exclusion criteria were: any change in maintenance COPD treatment in the previous 3 months; a previous diagnosis of asthma, sleep apnoea syndrome or chronic respiratory disease other than COPD; and any acute or chronic condition that would limit the patient’s ability to complete the questionnaires.

### Assessments

Night-time, early morning and daytime symptoms, severity of airflow obstruction, dyspnoea severity, health status, anxiety and depression levels, sleep quality and physical activity levels were assessed at baseline. COPD symptoms were assessed using a Night-time, Morning and Daytime Symptoms of COPD questionnaire developed by the study sponsor. To ensure accurate translation and a clear understanding of the questionnaire in each participating country, linguistic validation based on five patients per country was performed before use of the questionnaire in the study. The Night-time, Morning and Daytime Symptoms of COPD questionnaire is a 33-item questionnaire that asks the patient about the prevalence, frequency and severity of COPD symptoms during each part of the day during (i) the week before baseline and (ii) a typical week in the month before baseline (defined as a week the patient considers most usual for them in the previous month). In addition, the questionnaire also contained questions related to sleep disturbances, rescue medication, anxiety due to symptoms, limitation of activities due to symptoms and concentration levels. The questionnaire consists of three parts (one part for each period during the 24-hours) and includes 13 items for night-time symptoms, ten items for morning symptoms and ten items for daytime symptoms. Night-time corresponds to the time from when the patient goes to bed until they get out of bed to start the day; morning is the time from getting out of bed until approximately 11 am; and daytime is from approximately 11 am until the patient goes to bed. Patients were asked about the frequency of symptoms related to breathlessness, coughing, bringing up phlegm or mucus, chest tightness, chest congestion and wheezing during each period. Patients were also asked about the overall severity of their night-time, early morning and daytime symptoms during the last week; symptom severity was scored as 1 (no symptoms); 2 (mild); 3 (moderate); 4 (severe) or 5 (very severe).

Dyspnoea was assessed using the modified Medical Research Council (mMRC) scale [16] with patients assessing their perceived breathlessness on a scale of 0 (breathlessness with strenuous exercise) to 4 (too breathless to leave the house or breathless when dressing or undressing). Health status was assessed using the COPD Assessment Test (CAT; total score range 0–40, <10 indicates low impact, 10–20 medium impact, 21–30 high impact, >30 very high impact on health status) [17]. Self-perceived anxiety and depression levels were assessed using the Hospital Anxiety and Depression Scale [18-21] (HADS; total score range 0–21 where ≥8 indicates a probable diagnosis [22,23]). Sleep quality was assessed using the COPD and Asthma Sleep Impact Scale (CASIS) [24,25]. Patients assessed the frequency of a range of sleep problems on a scale of 1 (never) to 5 (very often; several items are reverse-scored); individual item scores were summed to give a total raw score, which was linearly transformed to total scale score (range 1–100); higher scores indicate greater sleep impairment. At baseline, patients were also assessed as being sedentary (does not perform any type of physical activity), moderately active (patient performs some type of exercise two or three times a week) and active (patient plays sports or exercises more than three times a week).

### Study outcomes

Primary endpoints in the study were the prevalence, severity, and inter-relationships for night-time, early morning and daytime symptoms at baseline (assessed based on the Night-time, Morning and Daytime Symptoms of COPD questionnaire). To further explore the relationship between 24-hour symptoms and other aspects of COPD that affect patients’ well-being, secondary endpoints included the relationship between night-time, early morning and daytime symptoms and severity of COPD, dyspnoea severity, health status, levels of anxiety and depression, sleep quality and physical activity levels.

### Statistical analyses

All analyses were performed using the full analysis set, which comprised all patients who fulfilled the eligibility criteria and who completed the Night-time, Morning and Daytime Symptoms of COPD questionnaire. The data were analysed using only the available data for each outcome. Descriptive data are reported as mean ± standard deviation (SD) or percentages, as appropriate. The relationship between symptoms in each part of the day and baseline characteristics or patient-reported outcomes was assessed using univariate analysis. The relationship between symptoms in each part of the day, symptoms and airflow limitation, symptoms and comorbidities and symptoms and physical activity level was assessed using a chi-squared test. The relationship between night-time, early morning or daytime symptoms and dyspnoea, health status, anxiety and depression levels, and sleep quality was assessed using a Wilcoxon rank sum test. All statistical tests were two-sided and used a 5% significance level; there was no adjustment for multiplicity. All statistical analyses were performed using SAS (version 9.1.3 or later; SAS Institute Inc., Cary, NC, USA).

A sample size of 680 patients offered a maximum margin of error (minimum precision) of 4% for estimating the percentage of patients with night-time, early morning and daytime symptoms, considering maximum indetermination (p = 50%) and a confidence level of 95%. Anticipating that approximately 5% of patients would have missing data or a major protocol violation, the final sample size was set at 720 patients.

## Results

### Patients

Of 743 patients who enrolled in the study and had a baseline visit, 727 were eligible for inclusion in the full analysis set. Demographics and baseline characteristics are shown in Table 1; 72.4% of patients had a diagnosis of moderate or severe COPD (based on severity of airflow limitation) and 58.9% had dyspnoea assessed on the mMRC scale as grade ≥2. Overall, 50.9% of patients were receiving treatment with triple therapy (long-acting β_2_-agonists [LABA], long-acting muscarinic antagonists [LAMA] plus inhaled corticosteroids), with or without a phosphodiesterase 4 (PDE4) inhibitor (2.1% and 48.8%, respectively). In addition to COPD, 79.4% of patients had a comorbid medical condition; 45.1% of patients had a diagnosis of hypertension and 33.7% had cardiovascular disease. Based on physical activity level, 30.0% of patients were assessed as being sedentary, 38.1% as moderately active and 31.4% as active at baseline.

**Table 1:** **Demographics and baseline characteristics**

**Characteristic**	**Eligible patients**
	**(N = 727)**
Sex, n (%), male	478 (65.8)
Age, mean (SD), years (n = 725)	67.2 (8.8)
BMI, mean (SD), kg/m^2^ (n = 720)	26.4 (5.2)
Current smoker, n (%)	202 (27.8)
Smoking history, mean (SD), pack-years (n = 723)	43.1 (24.8)
Post-bronchodilator FEV_1_, mean (SD), L (n = 696)	1.4 (0.6)
% predicted FEV_1_, mean (SD) (n = 718)	52.8 (20.5)
COPD severity, n (%)	
GOLD group I (mild)	63 (8.7)
GOLD group II (moderate)	265 (36.5)
GOLD group III (severe)	261 (35.9)
GOLD group IV (very severe)	73 (10.0)
mMRC grade, mean (SD)	1.8 (1.0)
mMRC dyspnoea grade, n (%)	
0	53 (7.3)
1	244 (33.6)
2	244 (33.6)
3	140 (19.3)
4	44 (6.1)
Patients with an exacerbation in previous year, n (%)	392 (53.9)
Number of COPD exacerbations in previous year, mean (SD) (n = 724)	1.2 (1.6)
Current COPD medication, n (%)^a^	
LABAs + LAMAs + ICS	355 (48.8)
LABAs + ICS	100 (13.8)
LABAs + LAMAs	70 (9.6)
LABAs alone	66 (9.1)
LAMAs alone	50 (6.9)
Short-acting bronchodilators^b^	22 (3.0)
LABAs + LAMAs + ICS + PDE4 inhibitor	15 (2.1)
LAMAs + ICS	8 (1.1)
Other^c^	19 (2.6)
No treatment	22 (3.0)
Total CAT score, mean (SD) (n = 721)	16.5 (8.1)
CAT score category, n (%)	
CAT score ≤10, n (%)	187 (25.7)
CAT score 11–20, n (%)	305 (42.0)
CAT score 21–30, n (%)	187 (25.7)
CAT score >30, n (%)	42 (5.8)
HADS anxiety score, mean (SD) (n = 710)	6.1 (4.2)
HADS depression score, mean (SD) (n = 714)	5.5 (4.1)
CASIS score, mean (SD) (n = 712)	44.1 (19.1)

n = patients with available data for each outcome; percentages are based on N = 727 patients.

^a^Used by >1% of patients.

^b^Includes: SABA alone; SABA + SAMA; SAMA alone.

^c^Includes: ICS alone; ICS + PDE4 inhibitor; LABA + ICS + PDE4 inhibitor; LAMA + LABA + PDE4 inhibitor.

BMI, body mass index; CASIS, COPD and Asthma Sleep Impact Scale; CAT, COPD Assessment Test; COPD, chronic obstructive pulmonary disease; FEV_1_, forced expiratory volume in 1 second; GOLD, Global Initiative for Chronic Obstructive Lung Disease; HADS, Hospital Anxiety and Depression Scale; ICS, inhaled corticosteroid; LABA, long-acting β_2_-agonist; LAMA, long-acting muscarinic antagonist; mMRC, modified Medical Research Council; PDE4, phosphodiesterase 4; SABA, short-acting β_2_ agonist; SAMA, short-acting muscarinic antagonist; SD, standard deviation.

### Prevalence and severity of COPD symptoms in each part of the 24-hour day

**Figure 1:**
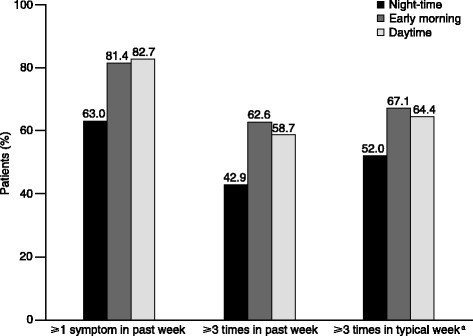
**Prevalence and frequency of night-time, early morning and daytime COPD symptoms (N = 727).** ^a^A typical week refers to a week that the patient considered most usual for them during the previous month. COPD, chronic obstructive pulmonary disease.

The prevalence of COPD symptoms in each part of the 24-hour day is shown in Figure 1. In each part of the 24-hour day, >60% of patients experienced at least one COPD symptom in the week before baseline (Figure 1). Early morning and daytime symptoms were most common, however 63.0% of patients experienced at least one night-time symptom in the week before baseline and more than half of the patients (52.0%) reported having night-time symptoms at least three times during a typical week.

Patients’ assessment of the severity of their night-time, early morning and daytime symptoms is shown in Table 2. In symptomatic patients the overall severity of symptoms was comparable for the night-time, early morning and daytime periods (Table 2). In each part of the 24-hour day, most people assessed their symptoms during the previous week as mild or moderate (night-time 89.5%, early morning 87.9% and daytime 89.3%).

**Table 2:** **Patients’ assessment of night-time, early morning and daytime symptom severity in the week before baseline**

**Symptom severity**	**No. of patients (%)**		
	**Night-time**	**Early morning**	**Daytime**
	**(n = 409** ^**a**^ **)**	**(n = 571** ^**a**^ **)**	**(n = 589** ^**a**^ **)**
Mild	191 (46.7)	252 (44.1)	254 (43.1)
Moderate	175 (42.8)	250 (43.8)	272 (46.2)
Severe	39 (9.5)	61 (10.7)	59 (10.0)
Very severe	4 (1.0)	8 (1.4)	4 (0.7)

^a^Patients who reported symptoms during the previous week and provided data for symptom severity.

COPD, chronic obstructive pulmonary disease.

### Individual COPD symptoms

**Figure 2:**
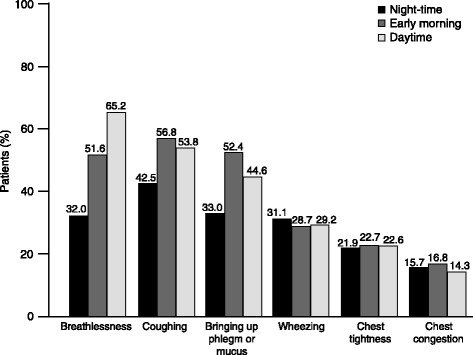
**Prevalence of individual COPD symptoms throughout the 24-hour day in the week before baseline (N = 727).** COPD, chronic obstructive pulmonary disease**.**

When individual symptoms were assessed, symptoms related to breathlessness were most common (71.4% of patients) followed by coughing (65.9%), bringing up phlegm or mucus (59.6%), wheezing (41.4%), chest tightness (32.9%) and chest congestion (23.4%). The frequency and pattern of each individual symptom varied throughout the 24-hour day (Figure 2). The proportion of patients reporting breathlessness increased from night-time through the morning and into the daytime, whereas coughing and bringing up phlegm or mucus were most common early in the morning. Coughing and bringing up phlegm or mucus were the most common symptoms reported during the night-time.

### Relationship between COPD symptoms in each part of the 24-hour day

**Figure 3:**
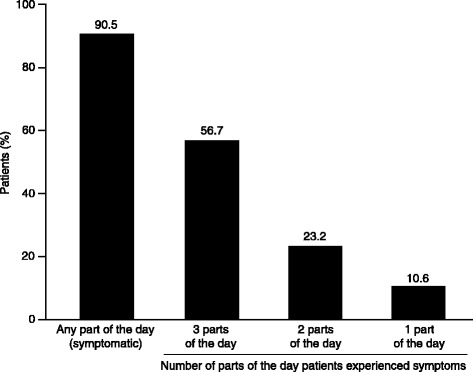
**Prevalence of COPD symptoms during one, two or three parts of the 24-hour day in the week before baseline (N = 727).** COPD, chronic obstructive pulmonary disease.

In the week before baseline, 90.5% of patients experienced COPD symptoms during at least one part of the 24-hour day (Figure 3). More than half of patients (56.7%) experienced symptoms throughout the whole 24-hour day; 10.6% of patients had symptoms in only one part (Figure 3). Almost 60% of patients had both night-time and early morning symptoms (Table 3). Among patients with night-time symptoms, 94.3% also had early morning symptoms while 73.3% of those with early morning symptoms also had night-time symptoms. A similar pattern was observed for the combinations of night-time and daytime symptoms (Table 3).

**Table 3:** **Proportion estimates of night-time, early morning and daytime COPD symptom combinations**

**Symptom combinations**	**% (n/N)**	**[95% CI]**
*Night-time (NT) and early morning (EM) symptoms*		
Overall patients with both NT and EM symptoms	59.8 (432/723)	56.1, 63.3
Patients with ≥1 NT symptom (n = 458) who also had ≥1 EM symptom	94.3 (432/458)	91.8, 96.1
Patients with ≥1 EM symptom (n = 589) who also had ≥1 NT symptom	73.3 (432/589)	69.6, 76.8
*Night-time (NT) and daytime (DT) symptoms*		
Overall patients with both DT and NT symptoms	59.4 (429/722)	55.8, 62.9
Patients with ≥1 NT symptom (n = 458) who also had ≥1 DT symptom	93.7 (429/458)	91.1, 95.6
Patients with ≥1 DT symptom (n = 598) who also had ≥1 NT symptom	71.7 (429/598)	68.0, 75.2
*Early morning (EM) and daytime (DT) symptoms*		
Overall patients with both EM and DT symptoms	75.0 (544/725)	71.7, 78.1
Patients with ≥1 EM symptom (n = 591) who also had ≥1 DT symptom	92.1 (544/591)	89.6, 94.0
Patients with ≥1 DT symptom (n = 601) who also had ≥1 EM symptom	90.5 (544/601)	87.9, 92.6

n = patients with available data for each combination.

When the relationships between symptoms during each part of the 24-hour day were assessed, there was a significant association for each potential symptom combination (night-time and early morning symptoms; night-time and daytime symptoms; and early morning and daytime symptoms; all p < 0.001). The relationships between night-time, early morning and daytime symptoms were maintained for all symptom combinations, irrespective of the severity of airflow limitation (mild to very severe all p < 0.05).

### Relationship between COPD symptoms in each part of the 24-hour day and other aspects of COPD

**Figure 4:**
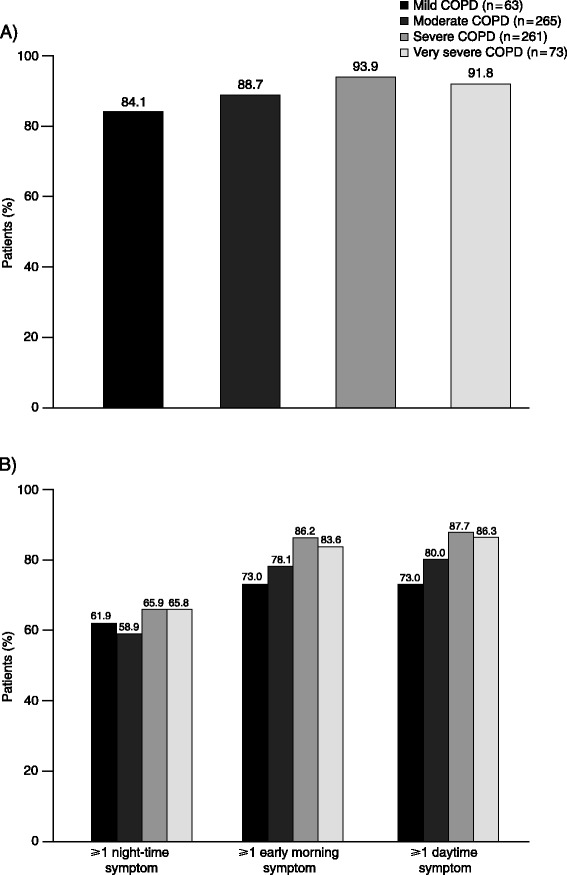
**Prevalence of any COPD symptoms (A) overall and (B) during each part of the 24-hour day, according to COPD severity.** n = patients in each group based on available data. COPD, chronic obstructive pulmonary disease.

The overall proportion of patients with any COPD symptom and the prevalence of night-time, early morning and daytime symptoms, according to COPD severity (based on airflow limitation) are shown in Figure 4A and B. Irrespective of the severity of airflow obstruction, >80% of patients in each severity category experienced COPD symptoms (Figure 4A). Overall, there was a significant relationship between COPD severity and symptoms during the early morning and the daytime (both p < 0.05). However, the relationship between night-time symptoms and COPD severity did not reach statistical significance and the proportion of patients with night-time symptoms was similar across all severities (58.9–65.9%; Figure 4B). Interestingly, there was also no significant relationship between COPD severity and the number of parts of the 24-hour day when patients experienced symptoms (p = 0.125); 47.6% of patients with mild COPD reported symptoms during the whole 24-hour day during the week before baseline compared with 63.0% of patients with very severe COPD.

There was a significant relationship between night-time, early morning and daytime symptoms and the severity of self-perceived dyspnoea (all p < 0.001; Table 4). Mean mMRC grades were significantly higher in patients with symptoms compared with patients without symptoms in each corresponding part of the 24-hour day (Table 4). There was also an association between the number of parts of the 24-hour day when patients experienced symptoms and dyspnoea severity (p < 0.001). Most patients who had grade ≥2 dyspnoea assessed on the mMRC dyspnoea scale had symptoms throughout the whole 24-hour day (63.8%); this compares with 46.5% of patients assessed as mMRC grade <2. There was no significant relationship between night-time, early morning or daytime symptoms and the presence of comorbidities in these patients.

**Table 4:** **Patient-reported outcomes in patients with/without COPD symptoms during each part of the 24-hour day**

	**Night-time symptoms**			**Early morning symptoms**			**Daytime symptoms**		
**Patient-reported outcome**	**No symptoms**	**≥1 symptom**	**p-value**	**No symptoms**	**≥1 symptom**	**p-value**	**No symptoms**	**≥1 symptom**	**p-value**
mMRC grade,	1.6	1.9	<0.001	1.4	1.9	<0.001	1.4	1.9	<0.001
mean (95% CI)	(1.5, 1.7)	(1.8, 2.0)		(1.2, 1.5)	(1.8, 2.0)		(1.2, 1.6)	(1.8, 2.0)	
	(n = 265)	(n = 457)		(n = 134)	(n = 591)		(n = 124)	(n = 600)	
CAT score,	11.6	19.3	<0.001	9.8	18.1	<0.001	10.0	17.9	<0.001
mean (95% CI)	(10.8, 12.4)	(18.6, 20.0)		(8.8, 10.9)	(17.4, 18.7)		(8.9, 11.1)	(17.3, 18.5)	
	(n = 263)	(n = 455)		(n = 133)	(n = 588)		(n = 124)	(n = 596)	
HADS anxiety score,	4.6	6.9	<0.001	4.1	6.5	<0.001	4.2	6.5	<0.001
mean (95% CI)	(4.1, 5.1)	(6.5, 7.3)		(3.4, 4.7)	(6.2, 6.9)		(3.5, 4.8)	(6.1, 6.8)	
	(n = 262)	(n = 445)		(n = 133)	(n = 577)		(n = 123)	(n = 586)	
HADS depression score,	4.2	6.2	<0.001	3.4	6.0	<0.001	3.7	5.9	<0.001
mean (95% CI)	(3.8, 4.6)	(5.8, 6.6)		(2.9, 4.0)	(5.6, 6.3)		(3.1, 4.4)	(5.5, 6.2)	
	(n = 263)	(n = 448)		(n = 132)	(n = 582)		(n = 123)	(n = 590)	
CASIS score,	33.6	50.2	<0.001	34.4	46.3	<0.001	34.2	46.2	<0.001
mean (95% CI)	(31.9, 35.2)	(48.4, 52.0)		(31.7, 37.1)	(44.8, 47.9)		(31.4, 37.0)	(44.7, 47.8)	
	(n = 260)	(n = 449)		(n = 131)	(n = 581)		(n = 122)	(n = 589)	

P values determined using Wilcoxon rank-sum test versus no symptoms in each period.

n = patients with available data for each outcome.

CASIS, COPD and Asthma Sleep Impact Scale; CAT, COPD Assessment Test; CI, confidence interval; COPD, chronic obstructive pulmonary disease; HADS, Hospital Anxiety and Depression Scale; mMRC, modified Medical Research Council.

In each part of the 24-hour day, including night-time, there was a significant relationship between symptoms and health status, anxiety and depression levels, and sleep quality (all p < 0.001 versus no symptoms; Table 4). In each period, mean CAT scores were >7.5 points higher in patients with symptoms versus patients without symptoms (Table 4). Based on HADS score at baseline, 34.5% of patients had anxiety and 27.6% had depression. When assessed according to patients with and without symptoms in each part of the 24-hour day, mean HADS anxiety and depression scores were significantly higher in patients with symptoms versus those without symptoms (p < 0.001 for all; Table 4). Sensitivity analyses performed in patients with no medical history of anxiety (n = 534) or depression (n = 538) also showed a significant association between symptoms and HADS anxiety and depression scores in each part of the 24-hour day (all p < 0.001). Patients with symptoms also had significantly higher CASIS scores compared with those without symptoms (p < 0.001 for all; Table 4), indicating greater sleep impairment. When patients who were receiving sleep medications or treatment for benign prostatic hyperplasia (n = 109) were excluded from the analyses of CASIS scores, the relationship between night-time, early morning and daytime symptoms and sleep quality remained significant in each period (data not shown).

In each part of the 24-hour day, there was a significant relationship between symptoms and patients’ physical activity level at baseline (assessed as sedentary, moderately active or active; p < 0.05 for each part of the 24-hour day). There was also a significant relationship for the number of parts of the 24-hour day when patients experienced symptoms and physical activity levels (p = 0.006). A higher proportion of patients who were sedentary had symptoms throughout the whole 24-hour day compared with patients who were active (64.2% versus 50.4%, respectively).

**Figure 5:**
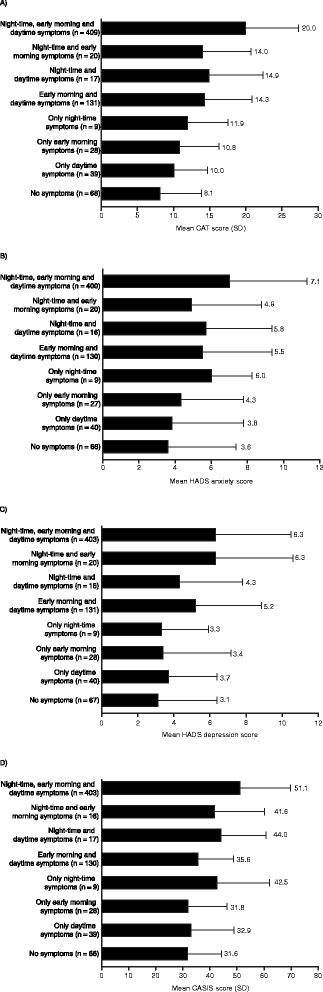
**(A) Health status, (B) anxiety, (C) depression and (D) sleep quality according to each combination of 24-hour COPD symptoms.** n = patients with available data for each outcome. CASIS, COPD and Asthma Sleep Impact Scale; CAT, COPD Assessment Test; COPD, chronic obstructive pulmonary disease; HADS, Hospital Anxiety and Depression Scale; SD, standard deviation.

Mean CAT, HADS anxiety and depression, and CASIS scores according to each 24-hour symptom combination are shown in Figure 5. Patients with symptoms throughout the whole 24-hour day had the worst health status and sleep quality and the highest levels of anxiety and depression. CAT scores were higher in patients with symptoms during two or more parts of the 24-hour day than in patients with only night-time, early morning or daytime symptoms (Figure 5A). With the exception of patients with early morning and daytime symptoms, patients who reported night-time symptoms, either alone or in combination, had the highest anxiety levels (Figure 5B) and patients with any combination of early morning and night-time symptoms had the highest depression levels (Figure 5C). A similar pattern was generally observed when HADS scores were analysed in patients with no medical history of anxiety or depression (Additional file 3). Patients with any night-time symptoms had worse sleep quality than patients without night-time symptoms (Figure 5D).

## Discussion

In this observational study, more than half of patients reported experiencing COPD symptoms throughout the whole 24-hour day, despite receiving ongoing treatment for their COPD and almost 80% of patients had symptoms during at least two parts of the 24-hour day. While early morning and daytime symptoms were most frequent, night-time symptoms were also very common and almost two-thirds of patients experienced at least one night-time symptom during the week before baseline. Importantly, any symptoms in the early morning, daytime or night-time were associated with worse outcomes across a range of patient-reported measures including more severe dyspnoea, higher anxiety and depression levels and worse health status and sleep quality.

The observation that a large majority of patients experienced symptoms during at least two parts of the 24-hour day is consistent with results from a recent real-world study in almost 1500 patients. The study by Roche et al. showed that most patients had symptoms during the daytime and night-time and only 34% of patients experienced COPD symptoms in isolation during one part of the 24-hour day [13]. However, in contrast to the results reported here, in the previous study daytime symptoms were by far the most prevalent (97% of patients) with just over one-third of patients reporting symptoms when getting up in the morning. This discrepancy may relate to the different definitions of morning symptoms used; the Roche et al. study defined morning symptoms as those present on waking and did not include those symptoms that persisted later in the morning [13].

There is no objective definition of ‘night-time symptoms’ in patients with COPD, and it has been suggested that night-time symptoms may be under-reported by physicians or may not be reported by patients [26]. The results of our study are consistent with a previous study in 2807 patients, which demonstrated that approximately 70% of patients reported experiencing night-time symptoms [7]. Together these data suggest a high prevalence of night-time symptoms in patients with COPD. Lung function exhibits circadian variation with reduced airflow during the night-time period [27]. The amplitude of this circadian variation has been shown to be increased in patients with COPD [28,29] and it may contribute to night-time symptoms [26,28]. In a previous study, wheezing was the most troublesome symptom at night, followed by cough [10]. In the present study cough and bringing up phlegm were the most prevalent night-time symptoms suggesting that, in addition to reduced airflow, other mechanisms may be involved in mediating night-time symptoms including mucus hypersecretion, reduced ciliary activity or increased cough sensitivity. Further investigation of these processes is required to better understand the pathophysiology underlying night-time COPD symptoms.

Early morning symptoms have been reported to be most problematic for patients with COPD and can significantly impact on daily activities [10-12] and working life [13]. Furthermore, in a previous study, a quarter of patients with COPD reported that night-time symptoms were most troublesome and night-time was the second most problematic time for patients with severe COPD [12]. However, despite patients frequently reporting night-time symptoms, the impact that symptoms at night has on daily activities, such as getting up for work, is often under-estimated by physicians [7]. Previous studies have shown a significant association between night-time symptoms and the severity of airflow obstruction in patients with COPD [7,14]. Interestingly, our results show that whilst there was a significant relationship between early morning and daytime symptoms and the severity of airflow limitation, this association was not significant for night-time symptoms and the prevalence of night-time symptoms was comparable across all severities of airflow limitation. Furthermore, symptoms in each part of the 24-hour day were inter-related, an observation that was consistent irrespective of COPD severity. These data suggest that the presence of night-time symptoms is not merely a consequence of more severe airflow limitation. Other mechanisms, such as decreased mucociliary clearance, could be involved. However, this study did not differentiate between different phenotypes of patients with COPD and further studies are required to determine if night-time symptoms are associated with a specific phenotype.

In each part of the 24-hour day, symptoms were associated with worse dyspnoea, health status, higher anxiety and depression levels, and greater sleep impairment. These are all outcomes that can impact on patients’ daily living and overall well-being. The difference in CAT scores between patients with and without symptoms in each period exceeded the estimated minimal clinically important difference (2 points) recently proposed [30], suggesting that symptoms in any part of the 24-hour day may be associated with a clinically meaningful worsening of health status. Moreover, anxiety and depression levels were also significantly higher in patients with symptoms compared with patients without symptoms. In general, anxiety levels tended to be highest in patients who had any combination of night-time symptoms and depression levels were highest in patients with any combination of night-time/early morning symptoms. Depression is a common comorbidity in patients with COPD [2] and patients with severe COPD have a 2.5-fold higher risk of depression compared with matched controls [31]. Comorbid depression is associated with an increased risk of exacerbation and mortality in patients with COPD [32]. Since symptoms of depression tend to be worse in the morning we cannot rule out that higher levels of depression contribute to night-time and morning COPD symptoms. Of note, examining questions on COPD symptoms and the HADS questionnaire does not reveal common items, making confounding by wording unlikely. Finally, a similar pattern in the magnitude of HADS scores and symptom combinations was observed in patients with no medical history of anxiety or depression. Sleep was also significantly impaired in patients with symptoms in any part of the 24-hour day compared with patients without symptoms. As expected, the greatest impairment was observed in patients with night-time symptoms. Poor sleep quality or sleep disturbance in patients with COPD has been shown to be associated with worse health status, more exacerbations, increased healthcare resource utilisation and increased mortality [7,33]. In this study, we also observed a significant relationship between symptoms in any part of the 24-hour day and physical activity levels: patients who were sedentary had more symptoms in each period than patients who were even moderately active. This may be important as low physical activity levels are significantly associated with poor quality of life and increased incidence of depression in patients with COPD [34] and have been shown to be a strong predictor of mortality in patients with COPD [35,36], and improving physical activity is an important goal in the treatment of COPD [9].

Overall, our results support previous studies showing that symptoms during the morning and the night-time are independently associated with worse outcomes in patients with COPD [7,13]. COPD symptoms when getting up in the morning have been shown to be independently associated with worse health status and more exacerbations, and have a negative impact on daily activities [13]. Similarly, patients with night-time symptoms had significantly worse breathlessness and health status and were more likely to have morning symptoms than patients without night-time symptoms, even when these analyses were controlled for confounding factors such as disease severity [7]. Our results extend these studies by demonstrating that there is an inter-relationship between symptoms in each part of the 24-hour day and that symptoms in any part of the day are associated with worse patient-reported outcomes.

While these results demonstrate significant relationships between symptoms in each part of the 24-hour day and various aspects of patients’ well-being, the analyses do not take into account confounding factors such as disease severity or comorbid conditions, which may also impact on patient-reported outcomes. Furthermore, no causal relationship can be inferred from the analyses as this was an observational study. Further investigation of the specific relationship between symptoms in each part of the 24-hour day and each outcome is required to establish whether symptoms are independently associated with the outcome, irrespective of underlying disease. While this study enrolled patients with mild to very severe COPD, only patients being treated in clinical practice (both primary care and specialist centres) were assessed. As such, the relevance of these observations for the wider population of patients with COPD, including those with undiagnosed COPD, requires further consideration.

## Conclusions

The results of this study demonstrate that despite receiving treatment for COPD, more than half of patients continued to have symptoms throughout the whole 24-hour day, including during the night-time and early morning periods. The relationship between night-time, early morning and daytime symptoms was observed irrespective of the severity of airflow obstruction. Patients with symptoms during any part of the 24-hour day also had significantly worse outcomes across a range of measures that impact on daily living, including health status, anxiety and depression levels and sleep quality compared with patients without symptoms. This suggests that current approaches to managing COPD may not adequately control symptoms, which can impact on a patient’s overall well-being. Newer therapies, including long-acting bronchodilators that are administered twice-daily or ultra-long-acting bronchodilators, may be useful in improving symptom control during the night-time, whereas those with a rapid onset of action may have advantages in controlling early morning symptoms. It is important for physicians to manage patients’ symptoms throughout the 24-hour day, even in those with mild airflow obstruction.

## Additional files

Additional file 1:
**ASSESS study investigators.** Details of study investigators. Additional file 2:
**Country approval authorities.** Details of approval authorities for each country. Additional file 3:
**(A) Anxiety and (B) depression according to each combination of 24-hour COPD symptoms in patients without anxiety/depression.** Analyses of HADS anxiety and depression scores in patients with no medical history of anxiety or depression.

## Abbreviations

BMI: Body mass index
CASIS: COPD and Asthma Sleep Impact Scale
CAT: COPD assessment test
COPD: Chronic obstructive pulmonary disease
FEV_1_: Forced expiratory volume in 1 second
GOLD: Global Initiative for Chronic Obstructive Lung Disease
HADS: Hospital Anxiety and Depression Score
ICS: Inhaled corticosteroids
LABA: Long-acting β_2_-agonist
LAMA: Long-acting muscarinic antagonist
mMRC: modified Medical Research Council
PDE4: Phosphodiesterase 4
SABA: Short-acting β_2_-agonist
SAMA: Short-acting muscarinic antagonist
SD: Standard deviation
