# Supplementary material for: Observational study to characterise 24-hour COPD symptoms and their relationship with patient-reported outcomes: results from the ASSESS study
Source: Respir Res. 2014 Oct 21;15(1):122. doi: 10.1186/s12931-014-0122-1 (PMC4220061; doi:10.1186/s12931-014-0122-1)
Supplement: Additional file 1: — ASSESS study investigators. Details of study investigators. [file 12931_2014_122_MOESM1_ESM.pdf]

Title: ASSESS study investigators

DENMARK: N Godtfredsen, Bispebjerg hospital, København NV; M Jensen, Regionhospitalet Viborg, Viborg; C Johnsen, Roskilde Hospital, Roskilde; C Nielsen, Aalborg Sygehus, Aalborg; P Tønnesen, Gentofte Hospital, Hellerup; FRANCE: A Bernardy, Polyclinique Cote basque Sud / Centre médical Arnasa, St Jean De Luz; P Burgel, Hôpital Cochin, Paris; D Caillaud, Hôpital Gabriel Montpied, Clermont-Ferrand; J Chavaillon, Centre Hospitalier d'Antibes Juan les Pins, Juan Les Pins; C Chouaid, Hôpital Saint Antoine, Paris; G Devouassoux, Hôpital de la Croix-Rousse, Lyon; D Lejay, Hôtel Dieu, Vieux Condé; D Piperno, Centre Medical P.A.R.O.T., Lyon; N Roche, Cochin Hospital, Paris; G Thabut, Hôpital Bichat Claude Bernard, Paris; S Verdier, Centre de Pneumologie, Perpignan; GERMANY: G Bourgeois , MVZ Dachau, Dachau; J Feimer , Praxis Dr. Feimer Pneumologie, Muenchen; C Franke , Facharztzentrum üBAG Sonneberg-Coburg, Sonneberg; C Geßner , Fachärzte für Innere Medizin und Bronchialheilkunde, Leipzig, Leipzig; T Ginko, Practice Dr. Ginko, Bonn; A Hellmann, Practice Dr. Hellman, Augsburg; F Herth, Thoraxklinik am Universitätsklinikum Heidelberg, Heidelberg; P Heymer, Klinische Forschung Dresden GmbH, Dresden; M Jandl, Hamburger Institut für Therapieforschung GmbH, Hamburg; A Linnhoff, Research Center for Medical studies (RCMS), Berlin; H Sudhoff, Lungenarzt Praxis Tegel, Berlin; C Vogelmeier, Klinik für Pneumologie at Universitätsklinikum Gießen und Marburg GmbH, Marburg; M Weber, Lungenpraxis Starnberg, Starnberg; T Welte, Med Hochschule Hannover, Hannover; H Worth, Klinikum Fürth, Fürth; ITALY: R, Antonelli-Incalzi, Policlinico Universitario Campus Biomedico, Rome; F Braido, Università degli Studi di Genova, A.O.U. S.Martino, Genova; M Carone, Istituto Scientifico di Riabilitazione di Cassano delle Murge, Cassano delle Murge; F De Bennedetto, Ospedale Policlinico Clinicizzato SS.Annunziata, Chieti Scalo; M Delucchi, Ospedale Civile di Saluzzo, Saluzzo; S Nardini, Ospedale di Vittorio Veneto-

ULS 7- Regione Veneto, Vitt. Veneto; A Pesci, Univ. degli Studi di Milano-Bicocca. Azienda Ospedaliera S.Gerardo, Monza; M Pistolesi, Ospedale Careggi di Firenze, Firenze; E Sabato, ASL Brindisi -P.O. "N. Melli" San Pietro Vernotico, San Pietro Vernotico; C Tantucci, University of Brescia - Spedali Civili di Brescia, Brescia; SPAIN: R Agüero, Hospital Marques de Valdecilla, Santander; E Balcells, Hospital del Mar, Barcelona; P Benavides, Hospital Universitario 12 de Octubre, Madrid; P De Lucas, Hospital Gregorio Marañón, Madrid; J Ferrer Sancho, Hospital Vall d'Hebron, Barcelona; J Gómez Seco, Hospital Universitario Fundación Jiménez Díaz, Madrid; J Hueto Pérez, Hospital Virgen del Camino, Pamplona; J Izquierdo Alonso, Hospital Universitario de Guadalajara, Guadalajara; P Marin, Hospital Cruces, Barakaldo; A Marin, Hospital Germans Trias i Pujol, Badalona; M Miravitlles, Hospital Vall Hebrón, Barcelona; E Monsó, Hospital Parc Taulí, Sabadell; J Sauleda, Hospital Universitario Son Espases, Palma de Mallorca; J Soler Cataluña, Hospital General de Requena, Requena; L Valdés Cuadrado, Hospital Universitario de Santiago, Santiago de Compostela; SWEDEN: D Curiac, Me3+ Clinical Trials, Göreborg; A Ekberg-Jansson, Angered Närsjukhus, Angered; A Lindberg, Sunderby Hospital, Luleå; C-G Löfdahl, Lund University Hospital, Lund; M Sköld, Karolinska University Hospital in Solna, Stockholm; A Sloma, Värmdö Vårdcentral, Gustavsberg;
THE NETHERLANDS: G-J Braunstahl, St. Franciscus Gasthuis, Rotterdam; M Deenstra, Flevoziekenhuis, Almere; R Djamin, Amphia Ziekenhuis, Breda; K Liesker, Tergooi Ziekenhuizen, Blaricum; C Melissant, Spaarne Ziekenhuis, Hoofddorp; T Molen, University of Groningen, Groningen; G Staaks, Meander Medisch Centrum, Amersfoort; P van Spiegel, Slotervaartziekenhuis, Amsterdam; UK: S Elamin, Nork, Bansted; D Halpin, Royal Devon and Exeter Hospital, Exeter; J Haughney, Glasgow Clinical Research Facility, Tennent Institute, Glasgow; L McGarvey, Belfast City Hospital, Belfast; A Mehta, Fairfield Surgery, Bookham; J Morjaria, Hull & East Yorkshire Hospitals NHS Trust, Cottingham; P Pearson,

Loncroft, Bansted; D Price, Research in Real Life Ltd, Cambridge; J Quint, University of London NHS Trust, London; C Roberts, Thorkhill Surgery, Thames Ditton; T Rogers, Doncaster Royal Infirmary, Doncaster; I Smith, Papworth Hospital NHS Foundation Trust, Cambridge; N Stevenson, Wirral University Teaching Hospital NHS Foundation Trust, Upton; W Wedzicha, Royal Free Hospital, London; A Wilson, Norfolk & Norwich University Hospitals NHS Foundation Trust, Norwich
