# Supplementary material for: Observational study to characterise 24-hour COPD symptoms and their relationship with patient-reported outcomes: results from the ASSESS study
Source: Respir Res. 2014 Oct 21;15(1):122. doi: 10.1186/s12931-014-0122-1 (PMC4220061; doi:10.1186/s12931-014-0122-1)
Supplement: Additional file 2: — Country approval authorities. Details of approval authorities for each country. [file 12931_2014_122_MOESM2_ESM.pdf]

Table. Country approval authorities

**Country**	**Approval authority**
Denmark	Danish Medicines Agency, Copenhagen, Denmark<br><br>Koncern Organisation og Personale – Region Hovestaden, Hillerød, Denmark
France	Conseil National de l'Ordre des Médecins (CNOM), Paris, France<br><br>Comité consultatif sur le traitement de l'information en matière de recherché dans le domaine de la santé (CCTIRS), Paris, France
Germany	Freiburger Ethik Komission International (FEKI), Freiburg in Breisgau, Germany
Italy	Comitato Etico of the Università Degli Studi "G. D'Annunzio", Chieti, Italy
The Netherlands	Centrale Commissie Mensgebonden Onderzoek (CCMO), Den Haag, The Netherlands
Spain	Agencia española de medicamentos y productos sanitarios, Madrid, Spain<br><br>Comité Ético de Investigación Clinica, Hospital Clínic de Barcelona, Barcelona, Spain
Sweden	Regionala Etikprövningsnämnden, Lund, Sweden
UK	National Research Ethics Service (NRES) Committee Yorkshire & The Humber – Leeds West, Jarrow, UK<br><br>Papworth Hospital Research and Development Unit (R&D Unit), Cambridge, UK
