# Supplementary material for: Observational study to characterise 24-hour COPD symptoms and their relationship with patient-reported outcomes: results from the ASSESS study
Source: Respir Res. 2014 Oct 21;15(1):122. doi: 10.1186/s12931-014-0122-1 (PMC4220061; doi:10.1186/s12931-014-0122-1)
Supplement: Additional file 3: — (A) Anxiety and (B) depression according to each combination of 24-hour COPD symptoms in patients without anxiety/depression. Analyses of HADS anxiety and depression scores in patients with no medical history of anxiety or depression. [file 12931_2014_122_MOESM3_ESM.pdf]

Figure. (A) Anxiety and (B) depression according to each combination of 24-hour COPD symptoms in patients without anxiety/depression

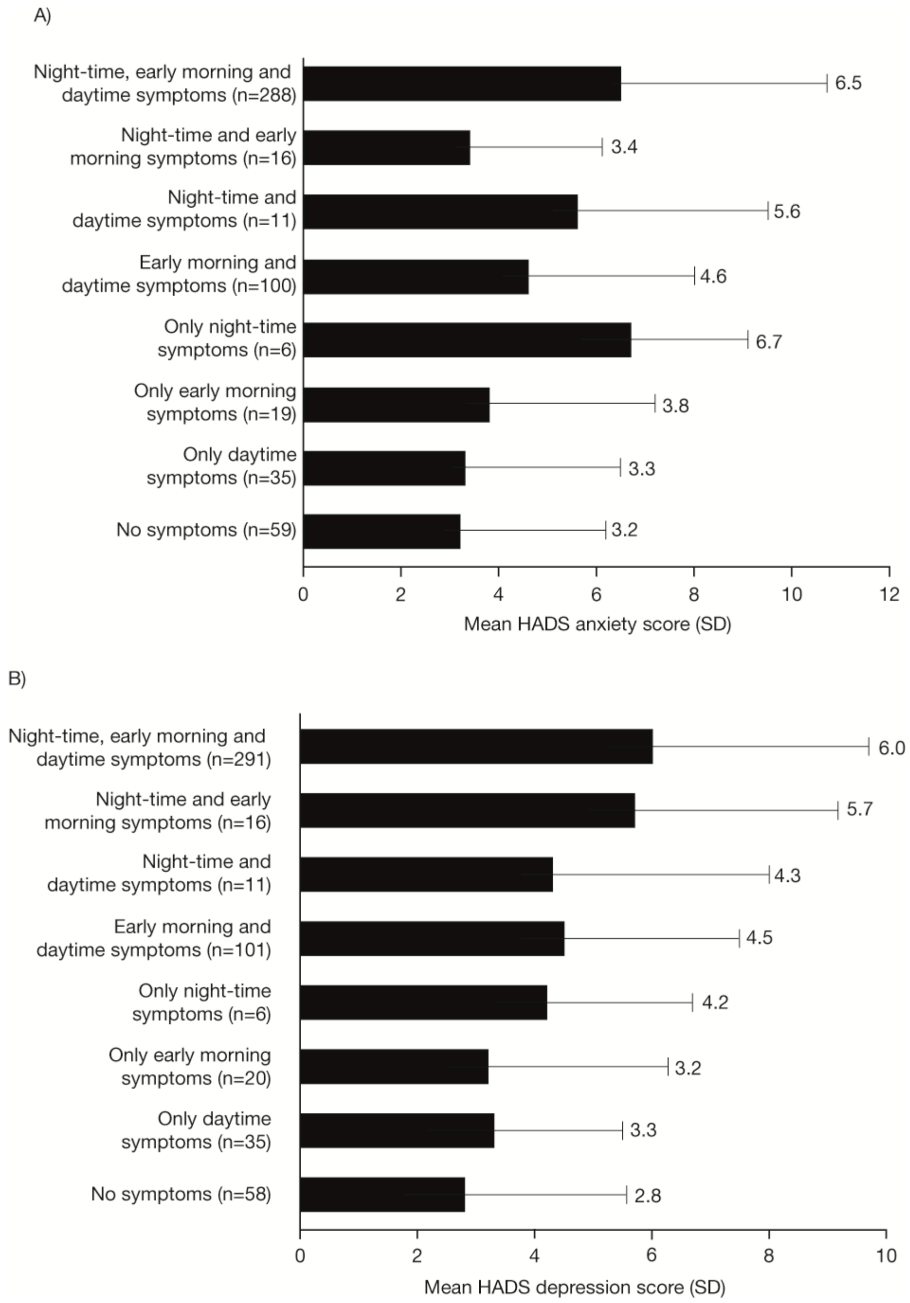

n=patients with available data for each outcome

Data are reported as mean ± SD

HADS, Hospital Anxiety and Depression Scale; SD, standard deviation
